# Editorial: Medicinal plants and their active constituents in the treatment of metabolic syndrome

**DOI:** 10.3389/fphar.2022.1031612

**Published:** 2022-10-17

**Authors:** Hui Teng, Lei Chen

**Affiliations:** Guangdong Provincial Key Laboratory of Aquatic Product Processing and Safety, Guangdong Province Engineering Laboratory for Marine Biological Products, Guangdong Provincial Engineering Technology Research Center of Seafood, Key Laboratory of Advanced Processing of Aquatic Product of Guangdong Higher Education Institution, College of Food Science and Technology, Guangdong Ocean University, Zhanjiang, China

**Keywords:** medicinal plants, metabolic syndrome, Alkaloids, pharmaceutical formulation, type 2 diabetes

This Research Topic in Frontiers in Pharmacology is dedicated to gathering new and creative work that covers the subject as thoroughly as possible. The work also provides insights into managing or solving complications related to metabolic syndrome (MS) using medicinal plants and their active constituents. MS is associated with increased risk of type 2 diabetes mellitus, non-alcoholic fatty liver disease, myocardial infarction, and stroke. Symptoms of MS can be managed by either pharmacologic interventions or supplementary treatments. Medicinal plants have been claimed to prevent MS, improve health, delay the aging process, increase life expectancy, and support bodily structure and function. Researchers have discovered numerous medicinal plants and have identified their active constituents (polyphenolics, alkaloid, terpenes, etc.); these have been suggested to alleviate MS and related complications through different mechanisms. Nowadays, natural products are vital for the discovery and development of numerous drugs, with significant implications for human and animal health. Plants have played a leading role as sources of phytochemicals of considerable nutritional and medical importance. Moreover, many other organisms such as marine organisms, terrestrial animals, and microorganisms produce molecules that are important drug candidates.

This Research Topic comprises six review papers that cover some of the ongoing research on phytochemicals. Several subjects such as *Tibetan Medicine for Diabetes Mellitus*, *Polygonatum in Prevention and Treatment of Diabetes*, and *Natural Products Targeting Liver X Receptors or Farnesoid X Receptor*, etc., are discussed. Most of these reviews describe the agronomic, botanical, phytochemical, and pharmacological features of selected plants that are used as food and/or medicines. The articles in this Research Topic have been viewed more than 1,500 times since 30th August 2022.

Yan et al. present a comprehensive overview of the mode and mechanism of action for various active constituents, extracts, preparations, and formulas used in Tibetan medicine. The dynamic, beneficial effects of these Tibetan medicine products are extensively discussed and summarized. These Tibetan medicines are valuable medical materials that can be developed into safe and effective agents for the management of DM and related complications. Gao et al. discuss the health-promoting effects of Ginsenoside Re, along with possible mechanisms of action to maintain good health. The authors summarize literature related to the pharmacological activity, pharmacokinetics, and toxicity of Ginsenoside Re, and provide a theoretical basis and guide for future Ginsenoside Re-related studies and applications (Gao et al.). In another review paper, the authors evaluate the mechanisms involved in the prevention and treatment of diabetes using Polygonatum. Three insulin signaling pathways which may be involved—p38MAPK, AMPK, and Wnt/β-catenin—are assessed (Liu et al.). Finally, and notably, hepatic mitochondrial dysfunction has been indicated to play an important role in the pathophysiology of non-alcoholic fatty liver disease (NAFLD). Alterations in the mitochondrial structure and function are observed in patients with metabolic syndrome; these alterations induce profound metabolic disturbances that contribute to the development of NAFLD. As such, Xu et al. review recent publications on this topic and reveal a relationship between mitochondrial function and the pathogenesis of NAFLD. The authors also summarize the protective effect of natural products (by improving mitochondrial homeostasis) against NAFLD and subsequent chronic liver diseases.

Several studies in this Research Topic illustrate the biological effects of phytochemicals such as polysaccharides obtained from Dictyophora (DIP). DIP pretreatment inhibits NaAsO2-induced L-02 cell apoptosis by increasing anti-apoptotic Bcl-2 expression and decreasing pro-apoptotic Bax expression (Hu et al.). Interestingly, by developing a toolbox of *in-vitro* assays and two *in-vivo* models, Günther et al. screened novel potent-plant extracts, derived from a plant extract library, against DM. The authors assess the antidiabetic activity of elephant grass using a diabetic rat model. The results indicate that *Cenchrus purpureus* extract efficiently ameliorates alloxan-induced pancreatic dysfunction, oxidative stress suppression, as well as inflammation and apoptosis *via* the activation of PI3K/AKT signaling pathways (Ojo et al.).

In recent decades, the role of the gut microbiota in altering host metabolism, thereby affecting metabolic syndrome, has garnered considerable research attention. In order to determine both the gut microbiota and metabolites related to glucose and lipid metabolic disturbances, Fang et al. perform metagenomic and metabolomic analysis on a group of normal mice, a group of mice with disturbances in glucose and lipid metabolism, and a group of mice with these disturbances present after berberine intervention. The authors found that the presence of berberine causes microbial and metabolic changes in the gut, which are well-correlated with improved glucose and lipid metabolism disturbances .

Finally, the positive effect of a traditional formula, namely, Sanghuang Tongxie (SHTXF) on an insulin resistance (IR) model is evaluated. The results reveal that SHTXF can activate phosphatidylinositol-3-kinase (PI3K) and can improve protein kinase B (PKB, or Akt) phosphorylation (Cao et al.). An alternative traditional Chinese medicine prescription, Huazhi-Rougan, is found to alleviate methionine-choline-deficiency (MCD) diet–induced non-alcoholic steatohepatitis in C57BL/6J mice. This is evidenced by an improvement in hepatic steatosis and inflammation, as well as a decrease in alanine and aspartate transaminases serum levels (Fang et al.).

The Editors wish to thank all Authors who contributed to this Research Topic with their ground-breaking work and novel ideas, as well as all colleagues who aided the review process. The Editors also warmly thank the Chief Editor of Frontiers in Pharmacology, Ethnopharmacology section, for the invitation to edit this issue and the opportunity to publish this compilation of articles. The support of the Guest Editors Dr Lei Chen, Qun Huang, Zhiling Yu, and John Thor Arnason is also thankfully acknowledged.

